# A qualitative study on the involvement of adolescents and young adults (AYAs) with cancer during multiple research phases: “plan, structure, and discuss”

**DOI:** 10.1186/s40900-022-00362-w

**Published:** 2022-07-08

**Authors:** Camila Rosalinde van Ham, Vivian Wilhelmina Gerarda Burgers, Sophia Helena Eva Sleeman, Annemiek Dickhout, Niels Christiaan Gerardus Laurus Harthoorn, Eveliene Manten-Horst, Mies Christina van Eenbergen, Olga Husson

**Affiliations:** 1 Department of Communication, Netherlands Comprehensive Cancer Organisation (IKNL), Utrecht, The Netherlands; 2 Department of Psychosocial Research and Epidemiology, Netherlands Cancer Institute, Amsterdam, The Netherlands; 3 Department of Medical Oncology, Netherlands Cancer Institute – Antoni van Leeuwenhoek, Amsterdam, The Netherlands; 4 Dutch AYA Care Network, Utrecht, The Netherlands; 5 AYA research partner, Amsterdam, The Netherlands; 6 Department of Medical Oncology, GROW – School for Oncology and Developmental Biology, Maastricht University Medical Centre, Maastricht, The Netherlands; 7 Department of Research and Development, Netherlands Comprehensive Cancer Organisation (IKNL), Utrecht, The Netherlands; 8 Division of Clinical Studies, Institute of Cancer Research, The Royal Marsden NHS Foundation Trust, London, UK; 9 Department of Surgical Oncology, Erasmus MC Cancer Institute, Erasmus University Medical Center, Rotterdam, The Netherlands

**Keywords:** Involvement, Collaboration, Adolescents and young adults, Involvement matrix, Cancer, Research phases

## Abstract

**Background:**

Including the lived experience of patients in research is important to improve the quality and outcomes of cancer studies. It is challenging to include adolescents and young adults (AYAs) cancer patients in studies and this accounts even more for AYAs with an uncertain and/or poor prognosis (UPCP). Little is known about involving these AYAs in scientific research. However, by including their lived experiences during multiple phases of research, the quality of the study improves and therefore also the healthcare and quality of life of this unique patient group. We first aimed to document experiences of AYAs and researchers with AYA involvement initiatives using the Involvement Matrix and the nine phases of the research cycle. Second, we aimed to map the (expected) challenges and recommendations, according to patients and researchers, for AYA involvement in each research phase.

**Methods:**

Thirteen semi-structured qualitative interviews were conducted with AYAs and researchers from February 2020 to May 2020. A thematic analysis codebook with a critical realistic framework was used to analyze the data.

**Results:**

AYAs and researchers were predominantly positive about AYA involvement within six of the nine phases of research: identify and prioritize topics, develop study design, disseminate information, implement, and evaluate findings. Not all respondents were positive about AYA involvement in the following three phases: formulate research questions, conduct research, and analysis and interpretation. However, few respondents had experience with AYA-researcher collaborations in multiple phases of the research cycle. Last, the results indicate the importance of adding a role (practical support) and two phases (grant application and recruitment) to the Involvement Matrix.

**Conclusion:**

Our results show the added value of AYA (with a UPCP) involvement within scientific research projects. We recommend researchers to actively think about the level and phase of collaboration prior to each research project, by involving and brainstorming with AYAs at the conception and throughout research projects. Besides, to enhance fruitful participation, we suggest thoroughly discussing the pros and cons of collaboration for each phase together with AYAs via the proposed Involvement Matrix to support transparency. We recommend to report experiences, choices, and results of AYA involvement.

**Supplementary Information:**

The online version contains supplementary material available at 10.1186/s40900-022-00362-w.

## Plain English Summary

Currently, more and more collaborations exist between patients and researchers in scientific research projects. Patient-research partnerships give patients the opportunity to add value to research by sharing their own experiences. Besides, patients themselves feel heard in their needs and are empowered. However, inclusion of young patients, like young adults with a cancer diagnosis, remains limited in research itself and specifically in the form of active involvement and collaboration initiatives. Thereby little is known about their age-specific problems and needs. This seems especially the case in young patient populations like adolescents and young adults (AYAs) with cancer who have a poor or uncertain prognosis. By analyzing the experiences of AYAs and researchers, this study aimed to unravel if collaboration within research projects is possible, and what defines the added value of such collaboration. Our study shows that researchers, as well as AYAs, think positively about collaborating during most phases of scientific research projects, even for challenging populations like AYAs with an uncertain and/or poor prognosis. Creating awareness of the possibilities of AYA involvement might increase involvement initiatives in future projects. This can be done using the Involvement Matrix. This is an existing tool which we further expanded according to our findings. Researchers and AYAs can fill in this tool (a table) together to plan, structure and discuss their collaboration. Our findings can be validated and might serve as a starting point for other patient groups.

**Supplementary Information:**

The online version contains supplementary material available at 10.1186/s40900-022-00362-w.

## Background

Nowadays, patient involvement in research is increasingly important and highly valued by funding agencies and academic journals in healthcare science [1]. In this study patient involvement does not only mean assistance with practical tasks, like preparing envelopes with printed questionnaires. It also, or even more, includes the application of patients’ experiential knowledge to initiate or adjust plans, activities, or discuss outcomes within a research project. For example, patients can bring unique perspectives from their ‘lived experience’ that researchers can use to answer more relevant research questions. This experiential knowledge of patients can improve recruitment strategies, contribute to more relevant study designs and a better translation and interpretation of the results to improve clinical practice [2–7].

Patient involvement might be useful within a unique population of cancer patients, namely adolescents and young adults (AYAs). Worldwide, different definitions exist of the AYA age range. In the US these AYAs are defined as young patients initially diagnosed with cancer between 15 and 39 years old [8]. In the Netherlands the age definition is set on 18–39 years due to specialized child oncology (0–18 years) [9]. AYAs face age-specific challenges like finding their (sexual) identity, building relationships, growing independence from parents and making important decisions about education, career and/or family [10].

Within this AYA population, initiatives of patient involvement have emerged in the past couple of years. Elsbernd et al. [11] developed a smartphone app together with AYAs with cancer, in which AYAs had an equally significant role as any of the professionals. In the Netherlands, ‘AYA Dreamteams’ were created in which experts and AYAs worked successfully together in equal value to define research questions, discuss ways to improve care, and conduct studies. Co-creation and co-design are fundamental goals of the Dutch AYA Care Network and can be used to build and achieve value based care [12]. AYAs might prefer a less paternalistic approach and instead be more inclined to be autonomous, in control, and involved within their own care trajectory compared to older generations [13]. The AYA population might therefore be an interesting patient group to further investigate the process of patient involvement and might be a main example for patient involvement in other patient populations.

Unfortunately, patient involvement initiatives remain limited in the population of AYAs with an uncertain and/or poor cancer prognosis (UPCP). These AYAs are diagnosed with advanced or metastatic cancer and will prematurely die from cancer since there is no reasonable hope of cure, but have no immediate threat of death. The life expectancy of these AYAs significantly improved due to new treatment options like personalized genotype-directed (immune) therapies. A recent study defined this patient group as AYAs with a UPCP. In this article this definition is used since it reflects the diversity and uncertainty within this population. AYAs with a UPCP experience prognostic uncertainty and constantly balance between concepts of hope and risks [14]. More (psychosocial) research is needed to examine the experiences of these AYAs to develop person-centered (long-term palliative) care for this group.

To improve the relevance, credibility, and value of research within this population collaboration with these AYAs might be of great value. This patient group could be seen as more challenging to involve within research given their constant uncertainty, unpredictable and fluctuating disease pattern, and shortened life expectancy [14]. Nevertheless, previous research showed it is feasible and valuable to involve these patients in research, even when the survival chances are low and end of life is near [1].

Although frameworks and guidance for involving patients at an organizational level have been published, patient involvement is often described as complex and context-dependent [15, 16]. To encourage sustainable and meaningful patient involvement, we need to agree on shared principles, purpose, level of inclusion (i.e. stages of research and/or the role of patients in each stage), processes, desired outcomes, and added value of patient experience. A framework developed to enhance meaningful patient involvement is the Involvement Matrix of Smits and colleagues [17], which divides research projects into three separate research phases: preparation, execution, and implementation (Table 1). For each of these phases the patients can fulfill different roles of participation (e.g. give advice to researchers or help in analyzing data). To guarantee successful participation, patients need to be involved in multiple research phases, and preferably early within the research cycle [18]. The Involvement Matrix can be a starting point for a dialogue between researchers and AYA cancer patients (with a UPCP) to examine which role the patient prefers within the research project [17].

**Table 1:**
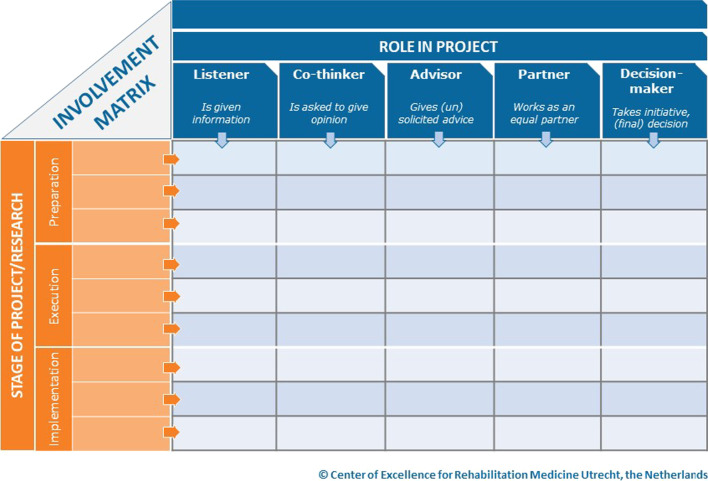
Involvement matrix

In the Involvement Matrix, five different roles for the patient can be distinguished for each stage of the project © Center of Excellence for Rehabilitation Medicine Utrecht, used with permission [17, 19]

**Fig. 1:**
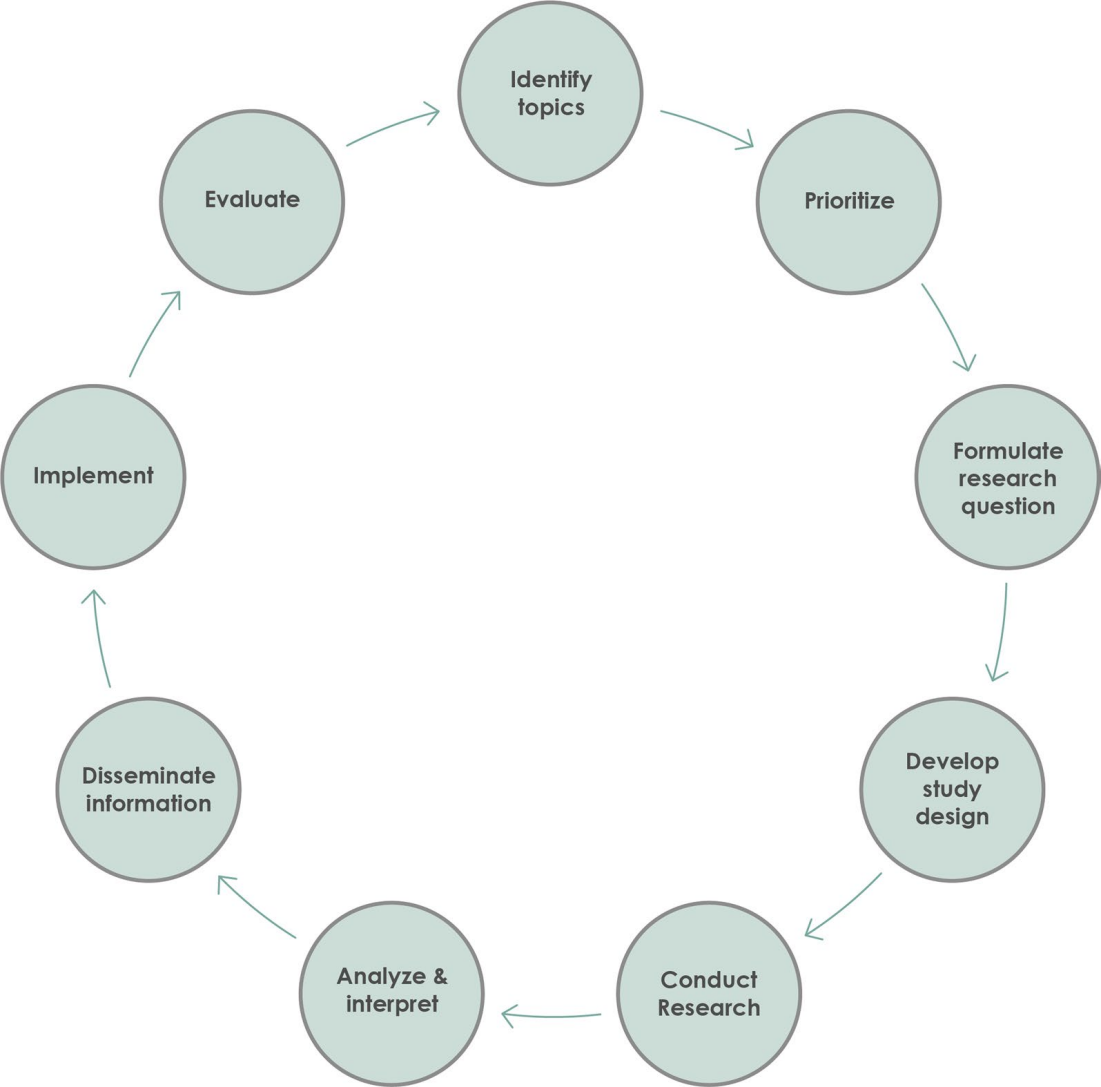
**Nine phases of a research project**. The nine phases of the research cycle are based on the visual of Vossen and Smit, used with permission [20]. More information and definitions of the phases can be found in Additional File 2

Today, most studies involve patients only at the first stages of research [1]. There is scarcity of well-described studies on how patients can participate in all phases of research. Besides, according to our knowledge, there is no research focusing on the process of involving AYAs with a UPCP during the research cycle. In order to develop relevant involvement initiatives for AYAs with a UPCP, it is useful to first examine the general process of AYA involvement during all phases of research (Fig. 1) via already existing experiences within the AYA population. We aimed to identify experiences of AYAs and researchers with AYA involvement initiatives using the models of Smits et al. [17] and Vossen and Smit [20]. In addition, the goal of this study was to map the (expected) challenges and recommendations, according to researchers and patients, for involvement of AYAs with a UPCP over the research cycle. This will provide transparent and valuable information on how to successfully involve patients in future research projects.

## Methods

A qualitative design, using semi-structured interviews, was used to explore the experiences, visions, and recommendations of AYA patients and researchers on the process of collaboration within (psycho)social AYA-research (IRBd20-205). A thematic analysis codebook with a critical realistic framework was used to analyze the data. This framework implies that each individual interprets reality in their own way, depending on their frame of reference [21]. To ensure quality we followed the COREQ-checklist [22].

### Study participants

All participants were recruited by email via a network of psychosocial oncology researchers (OH, VB). Criteria for selection of researchers were: previous, current, and/or future experience with AYA-participation in one or more phases of a (psycho)social research project. All AYAs were recruited via a larger study on AYAs with a UPCP, namely the COMPRAYA-study [23]. These AYAs with a UPCP were selected based on their past or current (and future) experience with involvement in research projects, in which they put their experiential knowledge into practice within a collaboration with researchers. As an exception, one AYA survivor without a UPCP was included, who was seen as a stakeholder due to relevant experience with involvement in research and the Dutch AYA Care Network. All three patients in this study had experiences with collaborations in scientific research project. Since only few AYAs with a UPCP and relevant experience could be included in this study, the results on this patient group are of an exploratory character.

### Data collection

For the interviews, two different interview guides for AYAs and researchers with open-ended questions and probes were created based on literature (see Additional file 1). The interview guides were adapted after four pilot interviews and four explorative conversations with AYAs and researchers, as well as discussions amongst the research team (VB; psychologist OH; epidemiologist and CvH; medical biologist with a specialization in participatory research (for more information see section Author information). Prior to the interviews the participants received a diagram of the research cycle (see Additional file 2), which was used during the interviews to structure the questions and experiences per research phase. The interviews lasted on average 64 min (range: 40–86 min). During the interviews additional questions were asked for a larger study, so the average duration of the interviews for this study were in fact shorter. During the interviews, several topics were addressed: participant’s experiences with AYA-collaborations, the exact activities and roles of the AYAs within each project, and a reflection on the (possible) advantages and/or obstacles of collaboration for each phase of the research cycle (Table 2). Since researchers had little to no experience with collaborations with AYAs with a UPCP, they were first asked to reflect on their collaborations with AYAs in general and were thereafter asked to reflect on the possible differences for AYAs with a UPCP (‘*What if..’ *type of questions). The semi-structured design of the interviews allowed participants to address new issues they believed to be relevant, whilst ensuring consistency amongst the different interviews. All interviews were conducted by CvH after participants signed an informed consent form. The interviews were held face to face, or by phone or video call due to COVID-19.

**Table 2:** Interview questions for AYA- and researcher participants

Questions researcher	Questions AYA
In which of the phase(s) of research did you collaborate with AYAs? *[showing the research cycle]*	Can you tell me something about your experiences with collaboration with researchers in scientific research? *If needed or unclear:* in which phases did you collaborate? *[showing the research cycle]*
What practical/concrete tasks did AYAs have during this collaboration? What was expected from them?	Which tasks did you have during the collaboration? Can you provide specific examples?
What did that actually look like in practice? *[asking for specific examples]*	What did you think about those tasks? What did you like? What did you not like?
Why did you choose to work together in this specific phase(s)?	
What were/are the difficulties with these phases (*constraining factors*)? And what went well (*enabling factors*)?*	What do you believe was pleasant about the collaboration? What could be points for improvement of the collaboration?
What do you think of AYA involvement in [*phase …*]? Why (not)? [*asking pros and cons per phase]*	What do you think of AYA involvement in [*phase …*]? Why (not)? [*asking pros and cons per phase]*
What do you think about involving AYAs in the entire research cycle?	What would your opinion be on being involved during all nine phases of research?
Does your opinion differ when it concerns involving AYAs with a uncertain or poor cancer prognosis? Why?	Does your opinion differ when it concerns involving AYAs with a uncertain or poor cancer prognosis? Why?
What do you think are difficulties when it concerns a collaboration with AYAs with life-limiting cancer? Why?	What do you think is important to keep in mind as a researcher when you want to collaborate with AYA patients? Are there differences when involving AYAs with life-limiting cancer? What could be constraining factors?

Some predetermined follow-up questions are included, some were dependent on the answers of the interviewee. * Known enabling and constraining factors on patient involvement from literature are shown in Additional file 1 of the Appendix

### Data analysis

All interviews were audiotaped, transcribed verbatim, and pseudonymized. During and after each interview, detailed notes were made on particularities and the interview process to provide the interview data with relevant context. Concurrent transcript reading and discussion with a member of the research team (VB) was performed to facilitate generation of new questions for subsequent interviews. Specific probes from previous interviews were used to confirm findings in subsequent interviews.

Data analysis was done by a thematic analysis codebook in ATLAS.ti 8.4.4 [24], using the six phases of inductive thematic analysis described by Braun and Clarke [21]. A codebook, based on the literature and interview guide, was used in which known obstacles and enabling factors for each research phase were summarized. In addition, an inductive orientation was used for each interview. This allowed for new themes to arise, for example, new advantages or disadvantages of collaboration for each phase. The analysis was an iterative process by which coding was performed by moving back and forth within the interview and between other interviews. The first interviews were coded by two researchers to promote trustworthiness (VB, CvH), the remaining data was coded by one researcher (CvH). During data analysis, notes were made of (initial) thoughts to structure and visualize codes, themes, and categories. Notes and codes were discussed with a researcher who works with AYAs with a UPCP (VB), to brainstorm ideas, to reflect assumptions, and check interpretations. The codes and themes were compared to findings from previous studies on patient involvement (e.g. advantages and disadvantages of collaboration).

In addition, a detailed Involvement Matrix was made based on plotting the nine phases of research from Vossen and Smit [20] against the five roles in the Involvement Matrix of Smits et al. [17] to document the current experience with collaboration of all participants. For each collaboration, the phase of research in which the collaboration took place was examined by the researcher (CvH) as well as the role of the patient based on the experience told by the participants. Where needed, additions were made to the Involvement Matrix to document all collaborations. The final manuscript was reviewed by AYAs as well as researchers. Some of them were research respondents to ensure correct translation of their views into the written results.

## Results

In this section, we first describe the demographics. These are followed by information on the collaborations of the participants with help of the Involvement Matrix (A). Next, we describe the advantages and disadvantages that participants mentioned for AYA-researcher collaborations for each phase of the research cycle (B), followed by specific challenges and recommendations that respondents mentioned for collaborations with AYAs with a UPCP (C).

### Demographics

Table 3 represents a summary of some characteristics of the respondents. In total twelve participants were included in analysis, of which nine academic scientists in the (psycho)social research domain (mean age = 34; SD = 10,5; range = 62–25) and three AYAs (mean age = 29; SD = 3,1; range = 32–25). First, four AYAs were included as participants but one interview with an AYA was incomplete and was therefore used as a pilot interview. Researchers were either PhD-students or (senior) post-docs, and their backgrounds included amongst others psychology, health sciences and epidemiology.

**Table 3:** Summary of the demographic information of researchers and AYAs

Participant	Sex	Age
Researcher	Female	62
Researcher	Female	32
Researcher	Male	32
Researcher	Female	29
Researcher	Female	25
Researcher	Female	27
Researcher	Female	35
Researcher	Female	27
Researcher	Female	35
AYA with a UPCP	Male	31
AYA	Female	25
AYA with a UPCP	Female	32

A. Phases and levels of collaboration

The majority of researchers had experience with AYA collaboration in one or more of the nine phases of the research cycle. The roles of the AYAs varied per phase from practical support to being in the lead of their own research (Table 4). Some researchers described that AYAs were partners in a phase, whilst follow-up questioning revealed that the actual role of the AYA was of a lower level (e.g. co-thinker). None of the researchers collaborated with AYAs in all phases within a single project. One researcher noted that seeing the research cycle made her think about collaborating in future phases as well. None of the researchers had experience with a research project concerning only AYAs with a UPCP. However, some mentioned collaborations where among others AYAs with a UPCP were involved. In addition, tasks were described which did not entirely match a role included in the Involvement Matrix or it was a combination of two roles. Some researchers reported collaborations where AYAs helped with practical tasks (e.g. posting questionnaires). Therefore, the role of “Practical support” was added to the Involvement Matrix during data analysis.

B. Examples of AYA involvement including pros and cons for each phase

**Table 4:** The roles of the AYAs in the collaborations based on the experiences reported by researchers

	Practical support	Listener	Co-thinker	Advisor	Partner	Decision-maker
Identify topics					| | |	|-
Prioritize				|*	| |	|-
Formulate research question			|	|		|-
Develop study design			| |	|* |*	|+	|-
Conduct research	| |				|+	|-
Analyze and interpret				|*	| |	|-
Disseminate information	|				|	|-
Implement						|-
Evaluate			|	|*		|-

The first column shows the nine phases of the research cycle [20]. The first row shows the five different roles AYAs could have based on the Involvement Matrix [17]. The role of 'practical support' was added, which encompasses AYAs helping with practical tasks like preparing questionnaire envelopes. Each line represents an example of AYA involvement mentioned by the participants of this study. The examples were classified into the Involvement Matrix. |- = One researcher reported that AYAs performed their own research under her supervision, which can be seen as a decision-maker role for all phases of their own research project. |* = role is not entirely clear but shows most aspects of advisor. |+ = role includes aspects of other roles e.g. providing feedback via email

### Phase 1 and 2: Identify topics and prioritize

Researchers mentioned that AYAs’ experiential knowledge is relevant when identifying research topics and prioritizing them since it can: lead to new insights, help to define relevant topics, and lead to a better nuance of topics that researchers already know from literature.

“You already know a lot of topics from literature, but not the nuances of it. What is for example the key element of the problem? You can only unravel this via talking about it with patients themselves.” Researcher

Collaborations within these phases were mostly (informal) focus groups, meetings (e.g. a brainstorm session with AYAs, researchers, and healthcare professionals) or one-on-one conversations, in which they discussed which topics are relevant. AYAs were positive about collaboration within these phases and felt that their experiential knowledge was useful. They reported that they felt their input was relevant and taken into account since it led to new follow-up discussions. However, an AYA mentioned that a somewhat predetermined framework is needed in the first phase to provide a starting point from which AYAs could share their opinions, otherwise it is hard for AYAs which input they need or want to share or where to start.

“Only saying: ‘We are going to brainstorm’ is too broad. I personally feel the need for a direction or framework. I want to know what is the overall topic or what do you want to discuss in the session. You don’t want to put words in their mouth, but you need a few scenarios and then see where the discussion goes.” AYA

### Phase 3: Formulate research question(s)

Most researchers and all the AYAs reported that the formulation of a research question is mostly the task of the researcher since it requires scientific knowledge. One researcher included AYAs in formulating research questions via online surveys. However, this resulted in a small number of usable research questions because patients were not familiar with the preconditions for scientific research questions. Nevertheless, she mentioned that the few usable research questions were unique.

“They come up with really good questions, which are still unanswered in research. These questions are truly formulated from the patient perspective, which I, as being a researcher, would never formulate but contains a very clear question.” Researcher

These questions were mostly formulated by highly educated patients and the researcher noted that this could indicate that lower educated AYAs might need guidance (from independent researchers) when it comes to involvement in this phase.

### Phase 4: Develop study design

Most researchers noted that collaboration when developing a study design is relevant, because AYAs have a better understanding of whether a certain design is suitable and feasible for the target group. One researcher mentioned that AYA involvement helped to refute worries of professionals during the development of an app.

“Oncologists had much worries about the possibility that AYAs would discover that some of the users of the app passed away and that this would lead to distress and fear. We brought back this worry to the AYAs. They told us they would also discover this without the app. They noted that indeed it is hard, but not harder than normal. Since they would otherwise hear it, for example, in the hospital that some peers did not make it. It is interesting that we can ask AYAs for advice when we feel resistance from oncologists.” Researcher

Another researcher reported that they did not collaborate in this phase during a survey study, which resulted in AYAs who rated the survey questions as irrelevant or hard to understand. Thereby she suggested that involving AYAs within this phase is important so you can make sure you ask the right and relevant questions, and to prevent that questions are interpreted differently than expected. However, some researchers reported that the substantive character of this phase may be an argument against collaboration. For example, this phase includes intellectual difficult tasks, which can result in the loss of low educated AYAs. AYAs mentioned that they can relevantly contribute to this phase and that it might even be the most important one to collaborate in, since the results of this phase (a chosen research method e.g. questionnaire) will continue to be used in the next phase.

“Collaborating in this phase is useful and maybe the most important. What is written on paper and decided at this point in time has to be correct, since this is what ends up with new patient participants in the next phase.” AYA

### Phase 5: Conduct research

Researchers’ opinions differed about collaboration in the phase of *conduct research*. One researcher believed that this phase requires scientific knowledge. However, another researcher successfully co-interviewed together with AYAs. She described that co-interviewing enhanced the dynamic of the conversation because of mutual recognition between the AYA and the AYA co-interviewer, which led to more efficient communication.

“Patient experts dare to ask much direct questions. [...] Where I sometimes try to talk around it a little bit, because I do not dare to go straight to it.. […] that girl sat down and said: “Well, how did you get your brain injury?” Researcher

This positive impact of co-interviewing was also reported by one AYA. According to this patient, you could build further upon statements of the peer and it could be worthwhile when the AYA gets stuck in wording during the interview. However, she mentioned worrying about a possible bias of AYA partners who co-interview, because they could steer the conversation. She mentioned that this might be reduced by providing AYAs with training.

“It [collaborating with an AYA] could result in a bias in this phase. An experience expert might be steering the conversation, which you [researcher] do not want. If you want to collaborate, I believe it is valuable to provide theme with a course. However, I believe it is valuable [to collaborate with AYAs] for example when the interviewee gets stuck in words.” AYA

One AYA felt no need to be a partner and would rather stick to only providing feedback when asked within this and the following phases of research, since he did not want to hear experiences of peers.

### Phase 6: Analyze and interpret

Some researchers argued that it requires scientific knowledge to analyze and interpret data, especially in quantitative research. Also for qualitative research it was argued that AYAs might lack relevant knowledge for the analysis. Mostly, they reported this encompassed for example that AYAs were not aware of the relevance to remain objective when analyzing qualitative data. One researcher collaborated in this phase and describes encountering this lack of objectivity among patients who had no additional training on qualitative data analysis, which led to results which did not meet the requirements of scientific research.

“We noticed that patients began to read the data which contained statements of the study respondents. I asked them if we should make a code of it, then they said: ‘No, because I do not agree with this statement’. That is very hard, because then you have to explain that you have to look at the data objectively. That is difficult, when you say: ‘This is not how it works’. Then they start thinking: ‘You ask me to think along but everything I say is wrong’. So there we needed to search for ways to analyze with patients without risking tokenism and not using their input.” Researcher

However, other researchers argued that collaboration might improve interpretation of data. One researcher provided AYAs beforehand with information about qualitative analysis, the data, preparatory questions and gave them time to delve into the data: “*so when they came in, they felt that they were quite prepared*”. This resulted into improvement in the interpretation of the qualitative data which led to relevant changes in the results, like redistribution of (sub)themes. AYAs argued whether the role of AYAs should be more at the background or might result in a bias, because they know the context of the results. Similar to the previous phase, it was argued that training might be helpful to prevent this bias.

### Phase 7: Disseminate information

All researchers reported that it is advantageous to collaborate when disseminating information of the study results.

“I have already done a duo presentation with [name] a few times. […] That is fantastic. [Name] talks about her own experience and that fits in nicely with the research I am doing, it is a very nice interaction.” Researcher

Some AYAs and researchers mentioned that when AYAs are involved in this phase, the final results can be disseminated to different populations in society, rather than only academics. Besides, when AYAs add their own experiences to the scientific results, it strengthens the key message since they can provide real examples. Lastly, AYAs and researchers mentioned that AYAs can help with improving the wording of the results, writing understandable non-scientific texts, and choosing a suitable medium to publish the results in. One researcher reported the desire to collaborate in this phase but mentioned that money is needed, e.g. for co-presenting with AYAs at (international) congresses.

### Phase 8: Implement

Most respondents described the importance of collaborations with AYAs during implementation because of the relevant experiential knowledge of AYAs, which can help to translate research findings into practical implications. Few noted that this phase is not specifically a task for AYAs. An AYA noted that it might be relevant to ensure that also low-educated AYAs are involved in this phase, as an innovation should be applicable to all patients.

### Phase 9: Evaluate

Most researchers and AYAs described the importance of involving AYAs within the evaluation. AYAs mentioned that it is important to include the opinions and feedback of AYAs in future research. Evaluating together with AYAs (within patient organizations) gives AYAs the chance to give feedback to the researchers on the participation process, by which researchers can learn and improve the way in which AYAs are being involved in future projects:

“I believe it is helpful to collaborate in this phase since AYAs can give feedback on the process like: ‘It was better if you have involved us earlier, or if you addressed a certain issue in another way the research would have run more smoothly.’ I believe it is valuable for researchers [to evaluate with AYAs] for your future research project.” AYA

### Additional phases: grant application and recruitment

Some researchers and AYAs mentioned two additional phases in which collaborations with AYAs are useful. At first, one AYA noted that AYAs can also be involved during *grant application*. She noted that the involvement of AYAs is often (only) at the very end of this phase in the form of a request for a letter of support, instead of involving patients at the conception of the study.

“As a researcher, you need to implement this into your time planning. And not only just before the deadline of a grant submission deadline. Often researchers realize just before the deadline of a grant application: I need to include some form of patient involvement. And then last minute start to ask patient organizations to help them out.” AYA

Secondly, the phase of *recruitment* was mentioned by most researchers and one AYA. Researchers noted that collaboration within this phase helps to recruit more study participants since AYAs can tell researchers how they themselves would most like to be recruited and how AYAs interpret researchers’ recruitment messages. These two additional phases and the additional AYA-role, mentioned in section A, led to an adjusted Involvement Matrix (Table 5).

C. Collaborating with AYAs with a UPCP

**Table 5:** Adjusted involvement matrix

	Practical support	Listener	Co-thinker	Advisor	Partner	Decision-maker
Identify topics						
Prioritize						
Formulate research question						
Develop study design						
Grant application						
Recruitment						
Conduct research						
Analyze and interpret						
Disseminate information						
Implement						
Evaluate						

The adjusted Involvement Matrix is based on the nine phases of research [20] and the Involvement Matrix with five roles [17]. Two phases, namely recruitment and grant application were added based on our findings, together with the additional role of practical support. A larger version of this table to fill in, can be found in Additional File 4

Similar as for AYAs in general, all AYAs and researchers believed that collaboration with AYAs with a UPCP is possible, even during multiple phases in research. However, respondents added some recommendations and challenges that were specific for this population. Some noted that flexibility was more important when collaborating with challenging populations like AYAs with a UPCP, because of their unpredictable disease pattern. This encompassed flexibility on the methods, frequency and tasks for AYAs, which requires extra input and energy from the researcher. Transparent communication was emphasized to discuss mutual expectations. Besides, they recommend to not confront AYAs with labels they do not know or do not identify with, e.g. ‘incurable’ while the AYA has a different interpretation of their medical status. AYAs reported that evaluating repeatedly on their preferences in collaboration during the phases of research is helpful and appreciated, since their health can vary over time. Since it is hard for researchers to estimate their health status, all AYAs mentioned that gauging and reminding were not experienced as overburdening.

“It is valuable to frequently speak to someone in a long-term palliative phase and to show interest. You have good versus bad moments. Your health and perspectives can differ a lot over time. In the beginning I reported that the cancer had no impact on my life, which was then indeed the case but right now it has [AYA was receiving radiotherapy and therefore was not able to work anymore]. The researcher with whom I collaborated said that it was useful to see how this can change over time.” AYA

Another aspect that researchers as well as AYAs mentioned was that researchers should use a personal approach when collaborating, which encompassed familiarizing with the medical condition of AYAs with a UPCP (e.g. what treatments are they undergoing and what are the side effects), and show empathy and engagement.

## Discussion

The results of our study indicated that both AYAs and researchers were predominantly positive about AYA involvement in six of the nine phases of research (identify and prioritize topics, develop study design, disseminate information, implement, and evaluate). The respondents were not overall positive in their thoughts and opinions about three phases: formulate research questions, conduct research, and analyze and interpret. This was mainly due to the perception that these phases required more scientific knowledge and skills, or their input was less relevant compared to the other phases. The opinions of AYAs and researchers did not show relevant differences. Besides, our study revealed that there are not many active AYA-researcher collaborations in multiple phases of the research cycle. Last, our results indicated the relevance of adding the role of practical support and two phases to the Involvement Matrix: recruitment and grant application.

The additional phases we found and suggest to implement in the Involvement Matrix, are in line with examples that were provided by Smits et al. in the manual of the Involvement Matrix [17, 19]. Other studies also showed the importance of the recruitment phase with successful involvement initiatives in cancer research during this phase [25, 26]. Filling in specific tasks in the Involvement Matrix and discussing it before the start of each research project might help to prevent tokenism since researchers are forced to actively think about the added value of AYA involvement and their possible roles within each phase of the project. Besides, the Involvement Matrix can create awareness among researchers on what patient involvement actually can look like in practice. Especially the addition of the role ‘practical support’ forces both parties to distinguish between practical tasks and more active involvement like partnership. Our study namely showed that researchers sometimes believe that AYAs fulfill higher roles of involvement (e.g. partnership), whilst in practice they had a lower role (e.g. co-thinker). This lack of awareness of what each role encompasses in daily practice, increases the risk that researchers believe they include qualitatively high levels of patient involvement whilst this is not the case. By filling in a detailed Involvement Matrix these risks for tokenism can be reduced by enlarging transparency. At last, also grant providers can use the tool to separate tokenism from valuable involvement in grant applications.

Remarkably, most of the respondents were positive about AYA involvement in most phases but they were not yet putting this into practice. During the interview the participants were asked to reflect on active collaboration in each phase, while showing them the research cycle, which resulted in a sense of realization of some respondents that collaboration is possible during many phases of research. Examples of successful collaborations with AYAs with cancer already exist [12]. This raises the question whether patient involvement is indeed complex or that researchers might think it is complex. In addition, researchers might not be aware of the possibilities to involve AYAs in a meaningful way in their research projects [27]. However, since various forms of involvement of patients exist and the interpretation of a ‘meaningful’ collaboration can differ per individual, it can be challenging to know where to start or how to perform ‘meaningful’ AYA involvement [7]. Besides, our study shows that the preferred role of AYAs within a project can differ throughout and between research projects, so there is not solely one best practice or way to involve patients [7]. The expanded Involvement Matrix can help to start a dialogue between patients and researchers during future research projects to give both parties a voice in the their preferences and expectations, whilst dealing with the fluid character of patient involvement.

Our respondents believed collaboration with AYAs was less relevant when formulating research questions. This is remarkable since studies show that patients are mostly involved during the development of cancer research including defining research questions [1, 28], and that young patients as well as researchers believe that young patients can come up with relevant research questions [7]. This might be explained by the fact that we split up the development of the research into three more specific phases: identify topics, prioritize topics and formulate research questions. Therefore, the phase of formulating research questions was in our study limited to transferring all previous input into a research question that meets up with scientific requirements.

Besides, some respondents were less positive about AYA collaboration during the conduct of research, and analysis and interpretation. However, most researchers who actually collaborated with AYAs within these phases were predominantly positive. This might suggest that researchers are not familiar with the possibilities of how to collaborate in a meaningful way within these phases rather than doubting the effectiveness [27, 29]. In previous studies some successful initiatives are described in which (young) patients were involved in multiple phases of research [30, 31], including data analysis [32–34]. However, still little involvement initiatives exist within cancer research during data collection [1].

Since researchers had little to no experience with collaborations with AYAs with a UPCP, they first reflected on their collaborations with AYAs in general and were thereafter asked to reflect on the possible differences for AYAs with a UPCP. Their opinions remained similar, though they added some recommendations which were specific for this population. Our results combined with previous studies suggest that involvement of challenging populations like (AYA-)patients in the palliative phase is possible, and that they are willing to collaborate with researchers [1]. Besides, young people like AYAs are motivated to be actively involved in research and contribute to society but do experience a lack of time as an obstacle [35]. Therefore, a precondition for these types of collaborations with palliative patients might be that they require more flexibility and specific measures, like resting time [1, 32, 36], since the disease pattern of AYAs with a UPCP can be unpredictable [14]. Besides, it might be interesting to further investigate if involvement of these AYAs in specific phases of research is valuable to spare time and energy for these patients. This since high level of engagement (like partnership) in key phases of research might be more feasible and valuable than only asking their opinion (like the role of ‘co-thinker’) during all phases of the project. Another solution for this might be to enlarge the pool of AYAs with a UPCP who want to collaborate, so that not each individual AYA needs to be involved in each phase of the project. A larger pool of AYAs might reduce pressure and expectations for individual AYAs, and researchers are less dependent on the availability of one AYA. By enlarging this pool, you can assess wishes and possibilities for each individual AYA to see what phase and role matches them best. AYAs with a UPCP are a relatively ‘new’ patient group with many undiscovered needs and problems, which in our opinion make them especially relevant to involve within scientific research, despite the specific challenges. The results for AYAs with a UPCP within this study show overlap with previous themes found in research on patient involvement, like the importance of transparent communication [6, 37, 38]. This shows that the results of this study might be useful to a variety of patient populations and future involvement initiatives.

The lack of skills and knowledge of AYAs as perceived by researchers as well as AYAs themselves was seen as a constraining factor for AYA involvement, which aligns with previous studies [39]. Our findings support training or additional explanation for patients to provide them with useful skills [1, 18, 20, 40], like remaining objective during qualitative data analysis. Our results show that the collaboration during analysis and interpretation was more successful when AYAs were provided with explanation beforehand. However, this might lead to the risk of losing AYAs due to increased time investment or workload. Therefore, we would recommend to only provide essential tools and information to prevent professionalization to maintain the valued differences between the scientific knowledge and skills of the researcher and the experiential knowledge of the AYA patient [29].

### Strengths

The qualitative design created the opportunity to examine challenges and recommendations of AYA involvement in much detail for each research phase. This study examined the involvement of underserved or underrepresented populations like AYAs with a UPCP [14]. This qualitative research is unique since it reflects on AYA involvement in general, rather than an evaluation of a specific project with AYA involvement. In addition, this study included AYAs as well as researchers, which strengthens the data since both perspectives are reflected. Last, AYAs and researchers were involved when defining the topic and scope of this research, the formation of the interview guide, and during the writing of the manuscript.

### Limitations

The experiences with collaborations described in this study are based on interviews rather than observations of the actual collaborations. We aimed to counterbalance the subjectivity by thoroughly asking follow-up questions during the interviews and to ask for concrete examples of the situation and tasks of the AYAs. Second, the small number of participants and the use of purposive sampling is a limitation, especially since less AYAs were included compared to researcher participants. The limited number of AYA participants requires therefore an exploratory interpretation of the results on AYAs with a UPCP. We therefore recommend future studies to further explore this topic with more AYA participants. However, limited researcher-AYA collaborations exist, especially with AYAs with a UPCP, which complicated the recruitment of more AYA patients. Nevertheless, via an extensive network of researchers we aimed to recruit the main players within the scope of this research. Finally, only one person did most of the coding, however, we aimed to include multiple perspectives in the analysis by discussing codes and all themes with another experienced qualitative researcher (VB).

### Practical implications

First, our study shows the importance of more awareness among researchers about the added value of collaborations with AYAs in the eleven phases of research. Second, tools like the Involvement Matrix might serve as a starting point for researchers to translate abstract concepts like ‘meaningful patient involvement’ into a concrete plan of action. They should be used already before the start of (AYA-)research projects, for example within the planning phase [30] to structure patient involvement [17], and discussed throughout the projects with involved patients (see Additional file 4 for an example of filled in Involvement Matrix). Third, providing researchers with training or additional support on these possibilities might help in creating awareness. This training might be provided by professionals in the domain of patient participation who can support the researchers in making a detailed participation plan by using the Involvement Matrix. Including patients in these trainings can be of added value to put knowledge directly into practice.

Besides researchers, also AYAs might benefit from additional support, especially when they are collaborating in the three phases which might require more skills (formulate research questions, conduct research, and analysis and interpretation). This might also enhance the confidence of AYAs. However, we would recommend to only provide essential tools and information to prevent professionalization and preserve valuable experiential knowledge [29].

In addition to recommendations on an individual level, we believe organizational changes are equally important. For example, more patient involvement might bring other obstacles already known from literature, like more time needed for recruitment of patients and resources for appreciation or involvement of patients [29]. This might implicate that a new way of thinking on patient involvement requires structural changes in research organization, e.g. structural subsidies for patient involvement or inclusion of obligatory and extensive participation plans in grant applications. The most important recommendations are summarized in Table 6.

**Table 6:** Highlights: the next steps to further improve AYA involvement

Highlights	Explanation
1. Create awareness	Researchers should be aware of the added value of collaborating with AYAs in multiple phases and/or levels of involvement during their research projects
2. Use tools	The adjusted Involvement Matrix is a valuable tool to plan, structure and discuss patient involvement
3. Provide training or support	Additional training or support should be available for researchers as well as AYAs to make valuable participation plans and/or learn skills which are helpful in AYA-researcher collaborations
4. Make structural changes	Structural changes are needed in the organization of research projects to overcome obstacles for collaboration already known from literature

The four most important recommendations of current study

## Conclusion

Our results reflect that it is of added value to collaborate and involve AYAs (with a UPCP) within scientific research projects. Our documentation of AYA involvement initiatives including specific pros and cons per research phase may help in structuring involvement in future research projects. We recommend researchers to actively think about the level and phase of collaboration prior to each research project, by involving and brainstorming with AYAs at the conception of research projects. Besides, to enhance fruitful participation, we suggest thoroughly discussing enabling and constraining factors, and preferences with AYAs via the proposed Involvement Matrix to support transparency. We recommend to report experiences, choices, and results of AYA involvement. This can provide future research projects with concrete examples and inspiration for involvement of (other) patients or other stakeholders.

## Supplementary Information

**Additional file 1**. Interview prompts.

**Additional file 2**. Research cycle.

**Additional file 3**. GRIPP2 short form.

**Additional file 4**. Adjusted involvement matrix A4-size.

## Data Availability

The datasets used and/or analyzed during the current study are available from the corresponding author on reasonable request.

## Abbreviations

AYA: Adolescent and Young Adult
UPCP: Uncertain and Poor Cancer Prognosis
