# Supplementary material for: A qualitative study on the involvement of adolescents and young adults (AYAs) with cancer during multiple research phases: “plan, structure, and discuss”
Source: Res Involv Engagem. 2022 Jul 8;8:30. doi: 10.1186/s40900-022-00362-w (PMC9264747; doi:10.1186/s40900-022-00362-w)
Supplement: Supplementary file 1 — Additional file 1. Interview prompts. [file 40900_2022_362_MOESM1_ESM.docx]

**Additional file 1: Interview prompts**

| **Enabling factors** | **Constraining factors** |
| --- | --- |
| 1. Suitable approach of patients 2. Using understandable language 3. Educate patients 4. Providing feedback & evaluation 5. Flexibility in methods 6. Eye for diversity (patients can choose their preferences role/task) 7. Atmosphere (trustful and safe, transparent and honest, respect, involvement) 8. Differences (language, power, knowledge) | 1. Tick boxing 2. No time 3. No money 4. Educate patients 5. Differences between patient and researcher (language, power, knowledge, values) 6. Recruitment is difficult 7. Dependent of other parties 8. Researcher does not see added value of patient involvement 9. Lack of skills (patient or researcher) 10. Overburdening patients |
| *Note: Some factors are in both columns since they were in some papers described as enabling and in some as constraining.* | |

*These prompts served as cues to which follow-up questions could be directed to. We always let the participants finish before asking these follow-up questions. The prompts are based on prior defined enabling and constraining factors from literature research on patient involvement (1-11).*

1. Boenink M, van der Scheer L, Garcia E, van der Burg S. Giving voice to patients: developing a discussion method to involve patients in translational research. NanoEthics. 2018;12(3):181-97.

2. Van der Scheer L, Garcia E, van der Laan AL, van der Burg S, Boenink M. The benefits of patient involvement for translational research. Health Care Analysis. 2017;25(3):225-41.

3. Caron-Flinterman JF, Broerse JE, Bunders JF. Patient partnership in decision-making on biomedical research: changing the network. Science, technology, & human values. 2007;32(3):339-68.

4. Bailey S, Boddy K, Briscoe S, Morris C. Involving disabled children and young people as partners in research: a systematic review. Child: care, health and development. 2015;41(4):505-14.

5. van Schelven F, Boeije H, Inhulsen M-B, Sattoe J, Rademakers J. “We know what we are talking about”: Experiences of young people with a chronic condition involved in a participatory youth panel and their perceived impact. Child Care in Practice. 2021;27(2):191-207.

6. Elsbernd A, Hjerming M, Visler C, Hjalgrim LL, Niemann CU, Boisen KA, et al. Using cocreation in the process of designing a smartphone app for adolescents and young adults with cancer: prototype development study. JMIR formative research. 2018;2(2):e9842.

7. Weston C, Soanes L, Chisholm J, Wiseman T. ‘Out There’: Developing a transition pathway for adolescents and young adults with cancer using Experience-Based Co-Design. The Journal of Health Design. 2018;3(1).

8. Elg M, Engström J, Witell L, Poksinska B. Co‐creation and learning in health‐care service development. Journal of Service Management. 2012.

9. Vossen C, Smit C. Handboek patiëntenparticipatie in wetenschappelijk onderzoek: ZonMw; 2006.

10. Abma T, Broerse JEW. Zeggenschap in wetenschap: patientenparticipatie in onderzoek: Lemma; 2007.

11. de Wit M, Bloemkolk D, Teunissen T, van Rensen A. Voorwaarden voor succesvolle betrokkenheid van patiënten/cliënten bij medisch wetenschappelijk onderzoek. Tijdschrift voor gezondheidswetenschappen. 2016;94(3):91-100.
