# Supplementary material for: A qualitative study on the involvement of adolescents and young adults (AYAs) with cancer during multiple research phases: “plan, structure, and discuss”
Source: Res Involv Engagem. 2022 Jul 8;8:30. doi: 10.1186/s40900-022-00362-w (PMC9264747; doi:10.1186/s40900-022-00362-w)
Supplement: Supplementary file 2 — Additional file 2. Research cycle. [file 40900_2022_362_MOESM2_ESM.docx]

**Additional file 2: Research cycle**


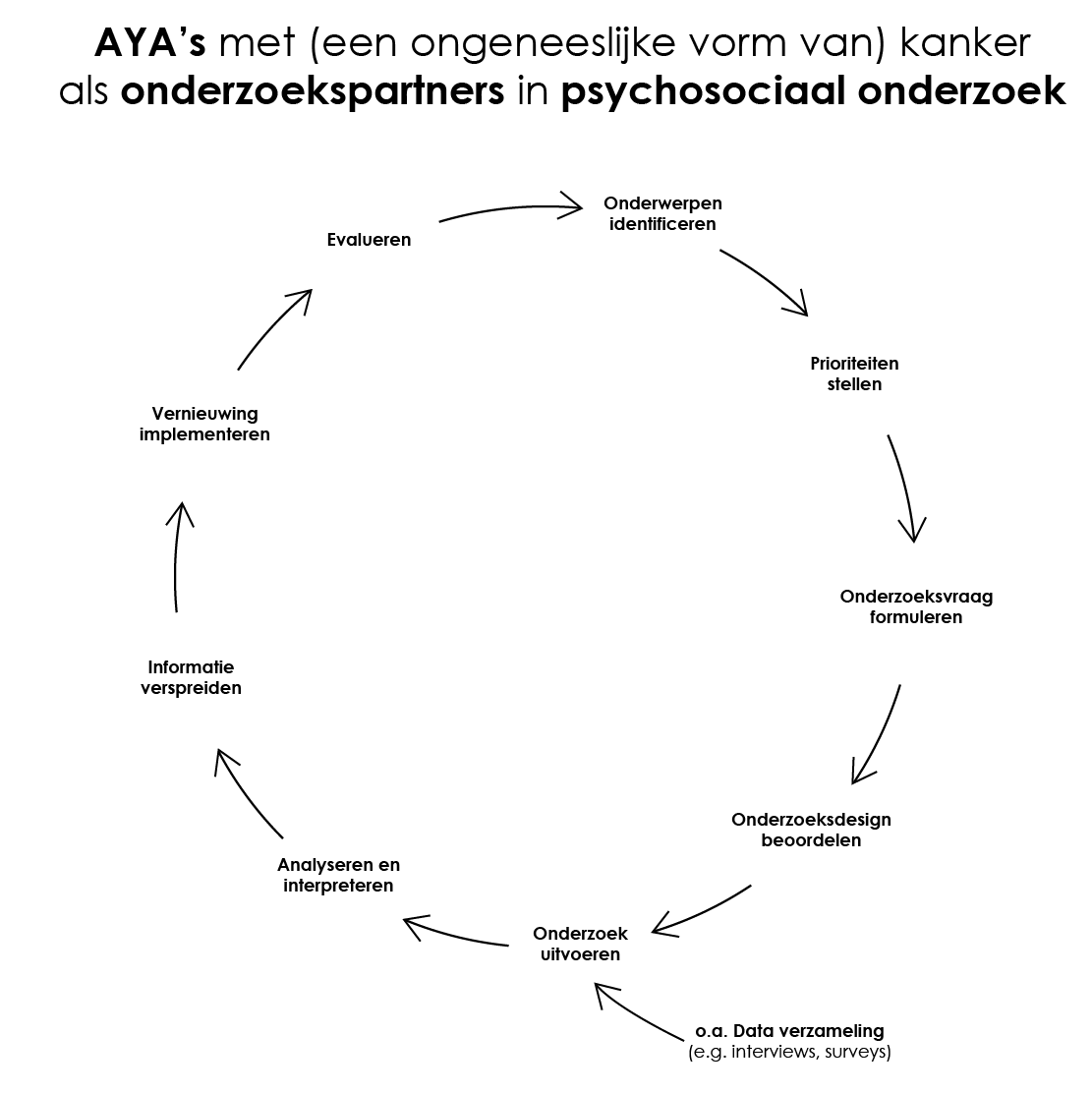
**
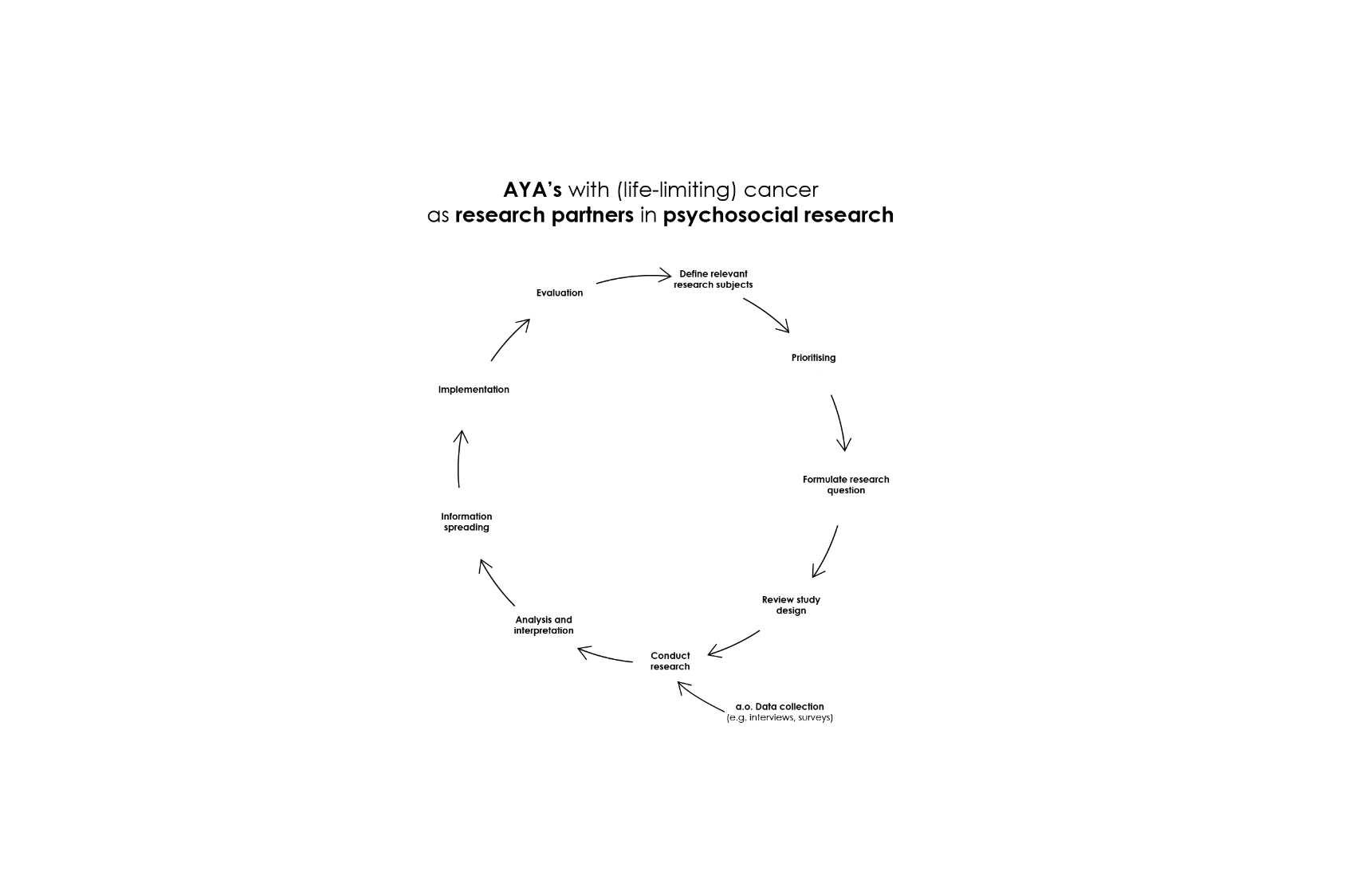
**

Dutch and English research cycle (18) that were given to the participants to discuss their experiences with collaborations, and pro’s and con’s for collaborating with AYAs in each phase. Phases were operationalized as followed:

1. Identify topics: Brainstorm about relevant research topics (possibly already within a predetermined scope).
2. Prioritize: Pick the most important topic(s) found in the previous phase.
3. Formulate research question: Transfer the chosen topic(s) into a scientific research question.
4. Develop study design: Choose the methods by which the data collection will take place, so e.g. interviews or questionnaires.
5. Conduct research: Carry out the chosen methods and collect data.
6. Analyse and interpret: Analyse and interpret the raw data to obtain results.
7. Disseminate information: Choose a medium and method to publish the results e.g. a presentation on a conference, a publication in a scientific paper or an article in non-scientific media.
8. Implement: When possible, transfer the results into an innovation which can be implemented, e.g. launch an app or introduce a new therapy into the healthcare system.
9. Evaluate: Discuss and evaluate the previous phases of research to define possible improvements, recommendations, and topics for future research.
