# Supplementary material for: A qualitative study on the involvement of adolescents and young adults (AYAs) with cancer during multiple research phases: “plan, structure, and discuss”
Source: Res Involv Engagem. 2022 Jul 8;8:30. doi: 10.1186/s40900-022-00362-w (PMC9264747; doi:10.1186/s40900-022-00362-w)
Supplement: Supplementary file 3 — Additional file 3. GRIPP2 short form. [file 40900_2022_362_MOESM3_ESM.docx]

**Additional file 3: GRIPP2 short form**

| Section and topic | Item | page No |
| --- | --- | --- |
| 1: Aim | **Report the aim of PPI in the study**: The aim of the study was to investigate current PPI initiatives in psychosocial cancer research with AYAs, and the challenges and obstacles related to this PPI amongst AYAs themselves and psychosocial researchers. | p. 4 |
| 2: Methods | **Provide a clear description of methods used for PPI in the study**: CvH had a dialogue with AYAs as well as researchers about the relevance of the aim and scope of this study. They were asked about their views on the topic of this study and the focus and set-up of the study was further defined using their input and experiences. Then, within the study semi-structured interviews were conducted to explore the experiences and views on AYA involvement of both researchers and AYAs on active collaboration within psychosocial research. Last, some respondents (AYAs and researchers) were asked to be co-authors to enhance correct interpretation of their input. | p. 5-6 |
| 3: Study Results | **Outcomes—Report the results of PPI in the study, including both positive and negative outcomes:** During the explorative conversations AYAs and researchers shared their views on the usefulness and relevance of the scope of the research (AYA involvement with a focus on AYAs with a UPCP). They also gave suggestion for who were relevant stakeholders to include as respondents within the study. The respondents within the study believed that it is relevant to involve AYAs within multiple phases of research. In addition, some specific challenges and recommendations were mentioned for each phase of research. Besides, both AYAs and researchers were positive on collaborations with more difficult populations like AYAs with a UPCP (see Result section of publication for a more detailed description of the interviews with AYAs and researchers). | p. 7-13 |
| 4: Discussion and conclusions | **Outcomes—Comment on the extent to which PPI influenced the study overall. Describe positive and negative effects:**  Patient and researcher involvement at the conception of this study helped in gaining a sense of an equal dialogue. Ideas and doubts about the scope were discussed with researchers as well as AYAs to test whether this research would indeed be relevant.  Asking AYAs and researchers on their views on and experiences with AYA-researcher collaborations during the study provided a starting point and concrete recommendations for future AYA involvement initiatives. We gave both parties of the collaboration a voice in sharing their preferences, experiences and views to hopefully enhance fruitful and relevant involvement initiatives in future projects.  Besides the explorative conversations and interviews, AYAs were asked to review this manuscript. This led to changes in the manuscript like wording, adding of (more) concrete examples, better and stronger translation of the research results into practical implications since they have relevant experiential knowledge on what kind of recommendations are useable in daily life and research practice. | p. 13-14 |
| 5: Reflections/critical perspective | **Comment critically on the study, reflecting on the things that went well and those that did not, so others can learn from this experience**: We wanted to involve more stakeholders, like AYAs, but since the scope was on participatory research rather than for example research on the specific needs and problems of the AYA population, it felt less useful. However, we believed including more AYAs for interviews would have been useful since the input of the AYAs was perceived as valuable since they were able to demonstrate abstract concept with examples in their daily life. This was also noted when AYA partners and researchers were asked to review this manuscript.  During the interviews we did notice that is was sometimes hard for AYAs to understand the concept of ‘research on research’, since the focus of this study was on methodology (how to involve AYAs) which could sometimes be abstract and difficult to reflect on. Despite, the current study adds relevant knowledge on AYA-involvement in psychosocial research and gives insights in possibilities to involve challenging populations. The study provides a helpful tool for researchers to start collaborating in their projects in the future. | p. 15-16 |
