# Supplementary material for: A qualitative study on the involvement of adolescents and young adults (AYAs) with cancer during multiple research phases: “plan, structure, and discuss”
Source: Res Involv Engagem. 2022 Jul 8;8:30. doi: 10.1186/s40900-022-00362-w (PMC9264747; doi:10.1186/s40900-022-00362-w)
Supplement: Supplementary file 4 — Additional file 4. Adjusted involvement matrix A4-size. [file 40900_2022_362_MOESM4_ESM.docx]

**Additional file 4: Adjusted Involvement Matrix A4-size**

|  | | **ROLE IN PROJECT** | | | | | |
| --- | --- | --- | --- | --- | --- | --- | --- |
|  |  | **Practical support**  *Helps with practical tasks* | **Listener**  *Is given*  *information* | **Co-thinker**  *Is asked to give opinion* | **Advisor**  *Gives (un)solicited advice* | **Partner**  *Works as an equal partner* | **Decision-maker**  *Takes initiatives, (final) decisions* |
| **PHASE OF PROJECT** | **Identify topics** | *1* |  |  | *2* |  |  |
|  | **Prioritize** |  |  |  |  |  |  |
|  | **Formulate research question** |  |  |  |  |  |  |
|  | **Develop study design** |  | *3* |  |  |  |  |
|  | **Grant application** |  |  |  |  |  |  |
|  | **Recruitment** | *4* |  |  |  |  |  |
|  | **Conduct research** |  |  |  |  | *5* |  |
|  | **Analyze and interpret** |  |  |  |  |  | *6* |
|  | **Disseminate information** |  |  | *7* |  |  |  |
|  | **Implement** |  |  |  |  |  |  |
|  | **Evaluate** |  |  |  |  |  |  |

*The Adjusted Involvement Matrix is based on the models of Smits et al. (17), and Smit and Vossen and Smit (20). This table can be used to give specific descriptions of the tasks which are in line with the different roles in each phase of research, including some examples. Each cell can be filled in with specific tasks/responsibilities of the patient. Some specific examples are described below and the correct field is depicted in the table with corresponding numbers:*

*1. Making notes of discussion; 2. Gives researcher (unasked) input for relevant research; 3. Receives final plan of chosen methods; 4. Mailing questionnaires to study participants; 5. Co-interviewer; 6. In the lead of analysis of interviewdata; 7. Gives opinion on a powerpoint presentation made of the research results for a conference.*
